# Curated Collection for Educators: Five Key Papers about the Flipped Classroom Methodology

**DOI:** 10.7759/cureus.1801

**Published:** 2017-10-25

**Authors:** Andrew King, Megan Boysen-Osborn, Robert Cooney, Jennifer Mitzman, Asit Misra, Jennifer Williams, Tina Dulani, Michael Gottlieb

**Affiliations:** 1 Emergency Medicine, The Ohio State University Wexner Medical Center; 2 Emergency Medicine, University of California at Irvine; 3 Emergency Medicine, Geisinger Medical Center; 4 Ohiohealth Learning, OhioHealth; 5 Emergency Medicine, University of Queensland; 6 Emergency Medicine, Hofstra North Shore-LIJ School of Medicine, North Shore University Hospital; 7 Department of Emergency Medicine, Rush University Medical Center

**Keywords:** curated collection, medical education, flipped classroom, pedagogy, modified delphi method

## Abstract

The flipped classroom (FC) pedagogy is becoming increasingly popular in medical education due to its appeal to the millennial learner and potential benefits in knowledge acquisition. Despite its popularity and effectiveness, the FC educational method is not without challenges. In this article, we identify and summarize several key papers relevant to medical educators interested in exploring the FC teaching methodology.

The authors identified an extensive list of papers relevant to FC pedagogy via online discussions within the Academic Life in Emergency Medicine (ALiEM) Faculty Incubator. This list was augmented by an open call on Twitter (utilizing the #meded, #FOAMed, and #flippedclassroom hashtags) yielding a list of 33 papers. We then conducted a three-round modified Delphi process within the authorship group, which included both junior and senior clinician educators, to identify the most impactful papers for educators interested in FC pedagogy.

The three-round modified Delphi process ranked all of the selected papers and selected the five most highly-rated papers for inclusion. The authorship group reviewed and summarized these papers with specific consideration given to their value to junior faculty educators and faculty developers interested in the flipped classroom approach.

The list of papers featured in this article serves as a key reading list for junior clinician educators and faculty developers interested in the flipped classroom technique. The associated commentaries contextualize the importance of these papers for medical educators aiming to optimize their understanding and implementation of the flipped classroom methodology in their teaching and through faculty development.

## Introduction and background

The flipped classroom (FC) learning model has become increasingly popular in medical education. In this educational pedagogy, learning materials are consumed independently in a learner-paced manner at home, while classroom time is devoted to knowledge application, simulation, case-based learning, or active discussion and problem-solving. Conversely, within a traditional classroom, foundational knowledge is passively transferred to learners through lectures delivered by instructors. Following the teaching session, learners actively use and apply the knowledge outside the educational setting [1].

Influenced by the preference of millennial learners for immediate, digital educational materials, medical educators are constantly searching for the most efficient and effective approach [2]. Active, self-directed learning is a necessary component of this learning model, and it is also a vital skill that provides the foundation for adult learning and continuing education. Social constructivism also provides a theoretical illustration of the benefits of the flipped classroom model [3]. Group collaboration encourages modelling, scaffolding, and feedback sharing, which engage learner preconceptions and build upon existing understanding [4]. This model promotes a higher level of learning, as defined by Bloom, which includes analysis, synthesis, and evaluation [4-6]. This has led an increasing number of educators to endorse the flipped classroom strategy in both the medical and primary education setting [7-9]. 

The Academic Life in Emergency Medicine (ALiEM) Faculty Incubator, an initiative developed in 2016, is an online community for practice and faculty development for early-career medical educators. Rather than encouraging the use of technology for novelty’s sake, educators in the Faculty Incubator aim at helping participants understand education theory, research, and program evaluation so that they can effectively utilize the most effective instructional design for meeting their teaching objectives. This integrative, narrative review was written to highlight the most important literature pertaining to the flipped classroom model for both junior and senior faculty educators.

## Review

Methods

Within the ALiEM Faculty Incubator, several scholarship groups were formed to focus on key topics of interest within medical education. The authors of this article selected the flipped classroom pedagogy as their topic. The group comprised four junior faculty members and four senior mentors. The online discussions within this virtual community involved junior faculty members and mentors. While the virtual discussions occurred, one author gathered all of the literature that was exchanged and recommended within the virtual platform and compiled the articles into a list. To ensure that a broad collection of articles was compiled, the authors augmented the collection with an open call for additional papers using Twitter with the hashtags #MedEd, #FOAMed, and #flippedclassroom and by contacting experts in the field. This query resulted in additional recommended papers that the experts identified as impactful via a PubMed search. The overarching purpose of this paper was to identify the top five recommended papers on flipped classroom pedagogy. It was not meant to provide an exhaustive list of all possible papers on the topic. 

The importance of these papers on flipped classroom pedagogy was evaluated through a three-round voting process inspired by the Delphi methodology, which has been previously described [10]. Each author read the 33 articles and actively participated in the entire process. In the first round, raters were instructed to indicate the importance of each article using a seven-point Likert scale, anchored at one by the statement “unimportant for junior faculty” and at seven by the statement “essential for junior faculty.” During the second round, raters were provided with a frequency histogram displaying how each article had been rated in the previous round. Participants were then asked to indicate if each article “must be included in the top papers” or “should not be included in the top papers.” In the third round, raters were provided with the results of the second round as the percentage of raters who indicated that each article must be included. Participants were subsequently instructed to select the five papers that were most important for inclusion in the article.

Similar methods were used by the ALiEM Faculty Incubator in a previous series of papers published in both the Western Journal of Emergency Medicine and Population Health [11-17] and Cureus [18]. This process does not satisfy traditional Delphi methodology [10] because the participants included novices (i.e., junior faculty participants in the ALiEM Faculty Incubator) as well as experienced medical educators (i.e., clinician educators, all of whom have published ten or more peer-reviewed publications and who serve as mentors and facilitators of the ALiEM Faculty Incubator). However, this was intentional, as the authors believe that the inclusion of more junior educators is essential to ensure that the selected papers would be of relevance to educators at different stages of their careers.

Results

Online discussions via the ALiEM Faculty Incubator in conjunction with social media calls yielded a total of 33 articles. The three-round modified Delphi process allowed our team to generate a rank-order listing of the included papers in order of perceived relevance, from the most to the least relevant. The top five papers are expanded upon below. Our ratings of all 33 papers are listed in Table 1, along with their citations.

**Table 1:** The complete list of flipped classroom literature collected by the authorship team

Citation	ROUND 1 Initial Mean Scores (SD) Max score 7	ROUND 2 % of Raters that Endorsed This Paper	ROUND 3 % of Raters that Endorsed This Paper in This Round	Top Five Papers
O'Flaherty J, et al. [19]	6.9 (0.4)	100%	100%	1st (tie)
Sharma N, et al. [20]	5.5 (1.2)	100%	100%	1st (tie)
Chen F, et al. [1]	5.8 (2.1)	87.5%	87.5%	3rd (tie)
Moffett J [21]	6.8 (0.7)	100%	87.5%	3rd (tie)
Jensen JL, et al. [22]	5.1 (1.8)	87.5%	75%	5th
Khanova J, et al. [23]	5.1 (1.0)	37.5%	0%	
Gross D, et al. [24]	5.1 (1.6)	87.5%	12.5%	
Betihavas V, et al. [25]	5.0 (0.9)	50.0%	0%	
Tan E, et al. [26]	4.9 (1.6)	75.0%	25%	
Mehta NB, et al. [27]	4.6 (1.4)	37.5%	0%	
Prober CG, et al. [7]	4.6 (0.7)	25.0%	12.5%	
Rose E, et al. [28]	4.6 (1.7)	50.0%	0%	
Mortensen CJ, et al. [29]	4.5 (1.9)	25.0%	0%	
Moraros J, et al. [30]	4.4 (2.0)	25.0%	0%	
Liebert CA, et al. [31]	4.3 (2.0)	62.5%	0%	
Sherbino J, et al. [5]	4.3 (1.2)	0%	0%	
Young TP, et al. [32]	4.3 (1.3)	12.5%	0%	
Morton DA, et al. [33]	4.1 (1.5)	12.5%	0%	
Riddell J, et al. [4]	4.1 (2.2)	25.0%	0%	
Wittich CM, et al. [34]	4.0 (1.9)	25.0%	0%	
McDonald K, et al. [35]	4.0 (0.8)	12.5%	0%	
Whillier S, et al. [36]	3.8 (1.3)	0%	0%	
Gilboy MB, et al. [37]	3.6 (0.9)	0%	0%	
Morgan H, et al. [8]	3.6 (1.6)	12.5%	0%	
Heitz C, et al. [38]	3.5 (1.5)	0%	0%	
Williams DE [39]	3.5 (1.2)	0%	0%	
Touchton M [40]	3.3 (1.7)	0%	0%	
Hanson J [41]	3.1 (1.2)	0%	0%	
Hsu SD, et al. [42]	3.0 (1.5)	0%	0%	
Tune JD, et al. [43]	3.0 (1.2)	0%	0%	
Missildine K, et al. [44]	2.9 (1.4)	0%	0%	
Periyakoil VS, et al. [45]	2.5 (1.3)	0%	0%	
Gubbiyappa KS, et al. [46]	2.4 (0.9)	0%	0%	

Discussion

The following is an annotated bibliography of the papers determined to be most relevant and valuable for junior faculty educators and faculty developers as determined by our modified Delphi process. The accompanying commentaries are intended to explain the relevance of these papers to junior faculty.

1. O'Flaherty J, Phillips C. The Use of Flipped Classrooms in Higher Education: A Scoping Review. Internet High Educ. 2015, 25:85-95 [19]

Summary: This review article provided an overview of the flipped classroom and its pedagogy and educational outcomes and identifies associated gaps. The fives stages of Arksey and O'Malley’s framework [47] form the basis of this review. The selected articles focussed on the types of resources, the pre-class and face-to-face phase, pedagogical acceptance by students and the faculty, design principles, and the evaluation outcomes of the flipped classroom approach. This review identified the risks and challenges associated with flipping the classroom, such as underutilization of a conceptual framework to build learning activities, lack of clarity and understanding in curricular design, and too much of a focus on content delivery. It also highlighted the potential of flipping the classroom as an opportunity for curriculum renewal and the development of a more student-centered approach. This review identified the need for stronger evidence in evaluating student learning and educational outcomes, resulting in improved critical thinking, problem-solving, and team management, as well as the need for guidelines about current approaches to assessment and feedback. The authors concluded that there is no single model for the flipped classroom currently, but core features include: providing content in advance, ensuring educator awareness of learner understanding, and focusing on higher-order learning during in-class time. 

Relevance to junior faculty members: O’Flaherty and Phillips discussed the current status of the flipped classroom pedagogy through a scoping review. They mentioned the structure, resources, implementation strategies, pedagogy, and educational outcomes, as well the gaps and challenges in implementing a flipped classroom. The authors suggested that online teaching material is good to teach lower-order cognitive skills. However, faculty should bear in mind the need to redesign the curriculum with active learning pedagogies to integrate pre-class activities with face-to-face classroom activities. Faculty members should utilize the flipped classroom pedagogy as an opportunity to redesign the curriculum to develop a more student-centric approach to increase critical thinking and learner engagement.

Considerations for faculty developers: Much of the published literature on the use of the flipped classroom has focused on primary education (i.e., K-12 learners). This scoping review provided insight into the current evidence supporting the flipped classroom in higher education. The review highlighted several issues of importance to faculty developers. As institutions push for more online education in response to changing learner demands, it will be critical to acknowledge the significant time investment needed for successful implementation. This review also discussed the “lack of pedagogical understanding of how to effectively translate the flipped classroom into practice” as a barrier to successful implementation [19]. Faculty development must focus on supporting faculty in developing skills for the design and implementation of the flipped classroom, including those related to determining learning objectives, using technology, lesson planning, and leading discussions.

2. Sharma N, Lau CS, Doherty I, et al. How We Flipped the Medical cCassroom. Med Teach. 2015, 37:327-30 [20]

Summary: This article provided a brief literature review on the flipped classroom pedagogy followed by a practical framework to be used by the educator when attempting to utilize the flipped classroom approach as a teaching modality. Sharma et al. began by outlining the common issues with lecturing, student information recall, and knowledge application. The flipped pedagogy classroom is an attempt to address these issues by allowing students to study abstract knowledge outside of the classroom and use the classroom as a means to apply the knowledge. The article then suggested a three-pronged approach - implementing, enacting, and evaluating - to flipping the classroom. These guidelines came out of the experience of flipping a rheumatology class at the University of Hong Kong. The authors offered advice to consider during the process of planning, implementing, and evaluating the flipped classroom. It is valuable to utilize a step-by-step approach over time with the goal of generating a well-planned curriculum that enhances student demand and motivation for adopting this classroom approach. Easily accessible media and online lesson plans that are engaging, and which are also quicker to review, were also recommended. Designing evaluations that target student depth of learning, satisfaction with curriculum design, and media use will help improve future implementations. In addition, reaching out and collaborating with faculty will provide further insight into improving flipped classrooms.

Relevance to junior faculty members: This article provided a much-needed framework for junior faculty who are beginning to think about the logistics and considerations of implementing the flipped classroom model. Often, overhauling an entire curriculum can be daunting and may be met with resistance from both faculty and students. This paper advised the reader to implement changes in a stepwise manner, ensuring that students understand the rationale behind the flipped classroom approach. It also recommended the use of different media to prevent boredom. However, it is also important to avoid complicated media and information overload. These pieces of advice are key to the novice educator trying to implement the flipped classroom model for the first time.

Considerations for faculty developers: Sharma and colleagues offered a pragmatic approach to implementing a flipped classroom [20]. Faculty developers tasked with training educators to implement a flipped classroom environment can utilize this article as a roadmap for similar curriculum design. Similar to O’Flaherty et al., this article also noted the importance of allocating sufficient time to plan the learning experience [19]. Additional advice was given about the design of the “pre-class” work, including the avoidance of information overload by using an appropriate lecture design. For “in class” work, they pointed out the importance of varied learning experiences to prevent boredom and improve student motivation, as well as the need for educators to recognize their changing role in the classroom. Consistent with other models for curriculum design [48], the importance of learner and program assessments was emphasized for the successful implementation of this curriculum.

3. Chen F, Lui AM, Martinelli SM. A Systematic Review of the Effectiveness of Flipped Classrooms in Medical Education. Med Educ. 2017, 51: 585-597 [1]

Summary: This article was a systematic review of the medical education literature on the flipped classroom method and evaluated claims of effectiveness. The systematic review process identified 46 articles for final analysis across 17 medical specialties. The articles related to both undergraduate and graduate medical education. Effectiveness was classified by Kirkpatrick’s method ranging from outcomes that change learner perception to those that affect patient outcomes. Lower-level outcomes, such as studies that demonstrated whether a student “liked” flipped classroom methods, were more prevalent than more rigorous studies, though the rigor did appear to increase over time. Results related to knowledge and skill acquisition were less robust. Overall, the authors of this paper concluded that the flipped classroom model was effective in terms of learner engagement and enjoyment, and was equivalent to traditional learning methods in terms of knowledge and skill acquisition.

Relevance to junior faculty members: This systematic review found that students reported a positive reaction to the flipped classroom approach. If faculty members desire to garner higher levels of engagement and interest, this can be one method to do so. Caution must be exercised to monitor for participation, as those students who do not participate do not demonstrate the same benefits. It is important for junior faculty to recognize this pitfall. Online preparatory work combined with an in-class assessment is an effective strategy. Given that higher levels of Kirkpatrick’s effectiveness have not been clearly demonstrated this method yet, faculty should be aware that the flipped classroom model may be part of a comprehensive educational strategy rather than the only method employed for knowledge and skill acquisition.

Considerations for faculty developers: Faculty developers must be familiar with a wide range of engaging instructional methods, including the flipped classroom. When teaching and mentoring faculty in this area, a faculty developer must understand the strengths and limitations of each instructional strategy. Although popular, according to this review, the flipped classroom does not evoke a significant improvement in knowledge acquisition, when compared to traditional methods. Learners do appear to enjoy the flipped classroom; thus, the flipped classroom achieves a positive “reaction” level in Kirkpatrick’s pyramid [49]. As the flipped classroom was only introduced to medical education in 2012 [1], there have been relatively fewer studies on the flipped classroom until recently. Future studies may demonstrate a larger effect than what has been shown in this systematic review. Therefore, faculty developers may encourage junior faculty members to engage in research related to the flipped classroom, especially with respect to graduate medical education.

4. Jensen JL, Kummer TA, d M Godoy PD. Improvements from a Flipped Classroom May Simply Be the Fruits of Active Learning. CBE Life Sci Educ. 2015, 14: ar5 [24]

Summary: This study compared a non-flipped, active learning model with a flipped classroom model, varying only the role of the instructor. Using a 5-E learning cycle model (Engage, Explore, Explain, Elaborate, and Evaluate) [50], in the non-flipped model, the instructor’s responsibility was in-class facilitation of content attainment, whereas in the flipped model, the student was responsible for content attainment prior to class. Two equivalent groups of 53 and 55 biology students from a private university in the United States were compared. There was no statistically significant difference in the final examination scores between the two groups. Both groups rated in-class facilitated activities as more influential on their learning than homework activities, suggesting that instructor support is important regardless of the phase of learning. Students in both groups lamented the absence of lectures although both groups identified the interactive nature of the course as their favorite characteristic. The study concluded that the flipped classroom model offered no additional benefit to student learning over a non-flipped, active learning approach.

Relevance to junior faculty members: The key point from this study is that students rate in-class activities as highly beneficial components of the course. The presence of a facilitator to support learning was seen as important, irrespective of the phase of learning during which the facilitator was present. Provision of individualized feedback and guidance during active learning and in-class activities were recommended. Peer interaction was also identified as a beneficial component of active learning models. Developing technologically-advanced supporting materials for a flipped classroom model was of no additional benefit over conducting an interactive course based on the principles of active learning. This finding justifies junior faculty focusing on developing their role as in-class learning facilitators and feedback providers, rather than spending time creating online content.

Considerations for faculty developers: Other papers in this primer suggested that the flipped classroom model does not improve knowledge retention when compared to traditional methods of instruction [1]. Similarly, Jensen et al. demonstrated no difference in performance between students who engaged in a flipped classroom and those who participated in an active, non-flipped classroom. Faculty developers must acknowledge, however, the many methodological flaws of published papers on the flipped classroom. First, the endpoints of many flipped classroom studies, including Jensen’s study, were written examinations, for which adequately valid or reliable evidence was rarely provided. Furthermore, the study participants in most of the studies were composed of a convenience sample. It is unclear if these studies could have detected more significant results in a larger sample size. After finding no difference in outcomes between students in a flipped classroom versus a non-flipped, active classroom, Jensen et al. concluded that the flipped classroom provided no additional benefit over other forms of engaging instruction. However, Jensen et al. only studied immediate recall and learner satisfaction as endpoints; there may be positives or negatives of the flipped classroom that were not observed in this study. Additionally, neither intervention was compared with a passive instruction control group, so it is unclear whether both groups provided any improvement in knowledge. Faculty developers must be made familiar with these flaws as they consume literature on the flipped classroom. Faculty developers must realize, however, that many interventions in medical education do not have strong supporting evidence.

5. Moffett J. Twelve Tips for “Flipping” the Classroom. Med Teach. 2015, 37: 331-336 [26]

Summary: The 12 tips defined in this article can be organized and summarized into three distinct themes. Prior to developing and implementing a flipped classroom curriculum, it is vital to utilize recognized educational theories and evidence-based techniques; understand the significant time, effort, and resources required; educate the learners on the pedagogy; and provide extensive faculty development for educators teaching with this method. As the curriculum is being implemented, educators should determine how to organize material; invest in the optimal pre-class materials; and utilize the flipped classroom methodology to tailor education to learner needs. Finally, educators must develop a method to evaluate their flipped classroom approach in order to provide necessary improvements and to further tailor the sessions to the needs of the learners.

Relevance to junior faculty members: This is a great primer for educators interested in implementing the flipped classroom pedagogical approach. The 12 tips provide salient advice and a model that is thorough, yet easily digestible, for junior educators. The article emphasized the importance of faculty development for educators prior to implementing sessions using this approach. Junior faculty educators are often enthusiastic and motivated. This article mentions the significant time and effort required to implement an effective flipped classroom curriculum. Similarly, a curricular flip does not have to be all or nothing, and sessions should be tailored to learner needs and desires. While the format of this paper presented a comprehensive review of the perils and pitfalls of flipping the classroom, the author also provided references for a deeper dive into these topics.

Considerations for faculty developers: Faculty developers will find this concise, clear paper helpful as both a reference for mentees, and in furthering their own understanding of the flipped classroom pedagogical model. In addition to foundational tips for flipping the classroom, the author highlighted some necessary concepts for faculty developers who mentor others in the execution of flipped classroom curricula. The author emphasized the importance of using sound educational theory in course design. Similarly, the author emphasized the importance of faculty development, time management, creativity, and flexibility in the implementation of the flipped classroom pedagogical approach. Program evaluation was also mentioned and represents another important consideration for faculty developers, especially since this aspect is frequently neglected by junior educators. Finally, the references included were thoughtful and relevant and would be good additions to both faculty developers’ and their mentees’ personal libraries.

Limitations

First, the selection of articles for the modified Delphi process did not incorporate an exhaustive, systematic search of the literature. Rather, the knowledge of experts in the field was combined with crowd-sourced feedback from the online community to generate the list of articles that were evaluated. The authors believe that this process was able to identify the key papers on the topic of interest while keeping the list a manageable length for the participants in the modified Delphi process. Given the process used, it is possible that some relevant articles may have been missed.

Similarly, in a pure application of the Delphi technique, all participants would be experts in faculty development. We declined this approach, opting instead to recognize the expertise of a range of faculty members. By incorporating junior faculty, we feel that we were better able to determine what is most relevant to that important cohort of medical educators.

Finally, the inclusion of authors within the field may have introduced bias in the selection of the articles. Considering the depth and breadth of our final list, we feel that by using these adjunctive methods, we have overcome any bias associated with our unstructured review of the literature.

## Conclusions

This paper describes some key papers that may be useful to both junior faculty educators and faculty developers interested in the flipped classroom pedagogy. We believe this resource will be valuable for clinician educators who seek to understand or implement a flipped classroom session or curriculum given both the rapidly evolving field of medical education and the associated needs of adult learners.
